# Cardiovascular Health at the Intersection of Race and Gender: Life-Course Processes to Reduce Health Disparities

**DOI:** 10.1093/geroni/igaa057.1635

**Published:** 2020-12-16

**Authors:** Chioun Lee, Soojin Park, Jennifer Boylan

**Affiliations:** 1 University of California, Riverside, Riverside, California, United States; 2 University of Colorado Denver, Denver, Colorado, United States

## Abstract

Objective: Higher cardiovascular health (CVH) scores are significantly associated with reductions in aging-related disease and mortality but racial minorities exhibit poor CVH. We examine the degree to which (a) disparities in CVH exist at the intersection of race and gender and (b) CVH disparities would be reduced if marginalized groups had the same levels of resources and adversities as privileged groups. Methods: We used biomarker subsamples from the Midlife in the United States (MIDUS) core study and Refresher studies (N=1,948). Causal decomposition analysis was implemented to test hypothetical interventions to equalize the distribution of early-life adversities (ELAs), perceived discrimination, or adult SES between marginalized and privileged groups. We conducted sensitivity analyses to determine to what degree unmeasured confounders would invalidate our findings. Results: White women have the highest CVH score, followed by White men, Black men, and Black women. Intervening on ELAs reduces the disparities: White men vs. Black women (30% of reduction) and White women vs. Black women (15%). Intervening on adult SES provides large disparity reductions: White men vs. Black men (79%), White men vs. Black women (70%), White women vs. Black men (25%), and White women vs. Black women (32%). Among these combinations, interventions on ELAs and adult SES are robust to unmeasured confounders. However, intervening on discrimination makes little change in initial disparities. Discussion: Economic security in midlife for Blacks helps reduce racial disparities in cardiovascular health. Preventing exposure to ELAs among Black women may reduce their vulnerability to cardiovascular disease, compared to Whites.

